# Conjugate of Thiol and Guanidyl Units with Oligoethylene Glycol Linkage for Manipulation of Oxidative Protein Folding

**DOI:** 10.3390/molecules26040879

**Published:** 2021-02-07

**Authors:** Shunsuke Okada, Motonori Matsusaki, Masaki Okumura, Takahiro Muraoka

**Affiliations:** 1 Department of Applied Chemistry, Graduate School of Engineering, Tokyo University of Agriculture and Technology, 2-24-16 Naka-cho, Koganei, Tokyo 184-8588, Japan; s-okada@st.go.tuat.ac.jp; 2 Frontier Research Institute for Interdisciplinary Sciences, Tohoku University, 6-3 Aramaki-Aza-Aoba, Aoba-ku, Sendai 980-8578, Japan; matsusaki@tohoku.ac.jp; 3 Institute of Global Innovation Research, Tokyo University of Agriculture and Technology, 3-8-1 Harumi-cho, Fuchu, Tokyo 183-8538, Japan

**Keywords:** oxidative protein folding, disulfide bond, thiol group, oligoethylene glycol

## Abstract

Oxidative protein folding is a biological process to obtain a native conformation of a protein through disulfide-bond formation between cysteine residues. In a cell, disulfide-catalysts such as protein disulfide isomerase promote the oxidative protein folding. Inspired by the active sites of the disulfide-catalysts, synthetic redox-active thiol compounds have been developed, which have shown significant promotion of the folding processes. In our previous study, coupling effects of a thiol group and guanidyl unit on the folding promotion were reported. Herein, we investigated the influences of a spacer between the thiol group and guanidyl unit. A conjugate between thiol and guanidyl units with a diethylene glycol spacer (GdnDEG-SH) showed lower folding promotion effect compared to the thiol–guanidyl conjugate without the spacer (GdnSH). Lower acidity and a more reductive property of the thiol group of GdnDEG-SH compared to those of GdnSH likely resulted in the reduced efficiency of the folding promotion. Thus, the spacer between the thiol and guanidyl groups is critical for the promotion of oxidative protein folding.

## 1. Introduction

**Figure 1:**
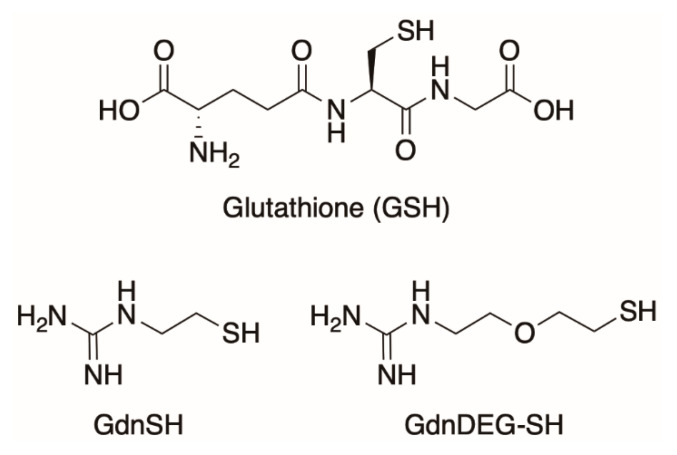
Molecular structures of glutathione (GSH), thiol–guanidyl conjugate without the spacer (GdnSH), and conjugate between thiol and guanidyl units with a diethylene glycol spacer (GdnDEG-SH).

Folding is an essential process of proteins to perform biological functions by forming the native conformation [1]. Defects in the folding cause formation of nonnative or misfolded proteins that are inactive or even pathogenic in connection to diseases such as Alzheimer disease, Parkinson disease, and diabetes [2,3,4]. In a cell, endoplasmic reticulum, responsible for the membrane and secretory protein folding, contains a number of chaperones and disulfide-isomerases that promote the disulfide-coupled protein folding, i.e., oxidative protein folding, to prevent protein aggregation [5]. Recent studies reported a link between the oxidative protein folding and the pathogenesis of misfolding diseases, including Alzheimer’s and amyotrophic lateral sclerosis [6,7]. Notably, protein disulfide isomerase (PDI) is a pathology-related enzyme involving in the oxidative folding of proteins, where the redox-active thiol groups in PDI play key roles for the effective protein folding promotion by cleavage, isomerization, and formation of the disulfide bonds during all folding stages of clients [7,8]. Inspired by the mechanisms by which PDI functions as a reductase or oxidase, PDI-mimetic synthetic molecules bearing thiol and selenol groups have been developed [9,10,11,12,13,14,15,16,17]. Recently, our group reported coupling effects of thiol and urea-type groups on the promotion of the oxidative protein folding [18]. Particularly, coupling of a thiol group and an iminourea (i.e., guanidyl) unit allows for increase in acidity and elevation of redox potential of the thiol group, which leads to a highly efficient folding promotion effect. Compared to glutathione (GSH), addition of GdnSH to reduced unfolded bovine pancreatic trypsin inhibitor (BPTI) accelerated the folding reaction to afford 2.1-times higher yield of the native form after 60 min incubation. Oxidative folding of ribonuclease A (RNase A) also proceeded promptly with an increased efficiency in the presence of GdnSH. In the presence of GSH, 34% enzymatic activity of reduced/denatured RNase A recovered after 180 min incubation. The recovery of native RNase A enhanced in the presence of GdnSH to afford 63% activity. In the present study, we developed GdnDEG-SH (Figure 1), a derivative of GdnSH bearing a diethylene glycol (DEG) spacer between the thiol and guanidyl groups, to investigate the effect of the spacer between the two functional units on the oxidative protein folding.

## 2. Results

### 2.1. Synthesis of GdnDEG-SH

**Figure 2:**
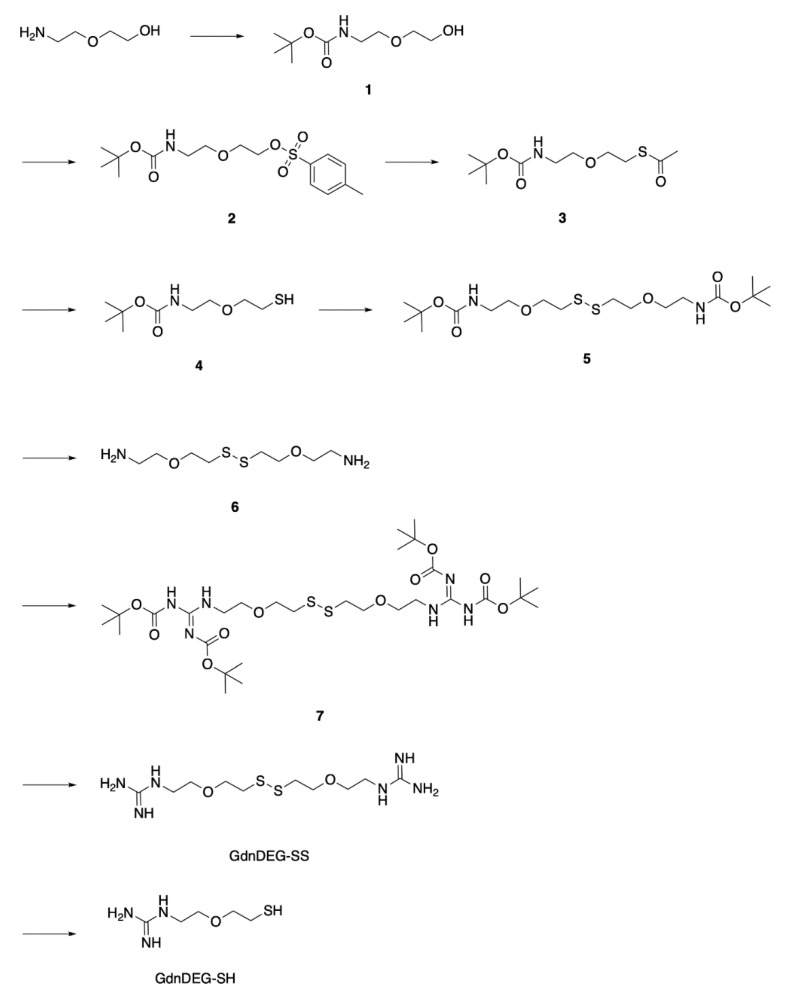
Synthetic scheme of GdnDEG-SH.

To study the spacer effect between the thiol and guanidyl groups, DEG was chosen as the spacer due to the water solubility with a neutral structure. To synthesize GdnDEG-SH, the amino group of 2-(2-aminoethoxy)ethanol was protected with *tert*-butoxycarbonyl (Boc) group by the reaction with (Boc)_2_O in EtOH to afford compound **1** in 94% yield (Figure 2). The hydroxy group of **1** was tosylated with TsCl in the presence of NaOH to afford compound **2** in 86% yield. The tosyl group in **2** was converted into a thioacetyl group to provide compound **3** in 92% yield by the reaction with AcSK in DMF. Hydrolysis of the acetyl group of **3** with K_2_CO_3_ afforded compound **4** in 42% yield, which was then converted into its disulfide form **5** in 80% yield by the oxidation with H_2_O_2_ and NaI in AcOEt. Deprotection of the Boc groups of compound **5** with trifluoroacetic acid (TFA) in CH_2_Cl_2_ afforded compound **6** in 99% yield. In order to introduce the guanidyl units, compound **6** was reacted with *N*,*N*′-bis(*tert*-butoxycarbonyl)-1*H*-pyrazole-1-carboxamidine in DMF in the presence of triethylamine to provide compound **7** in 66% yield. Deprotection of the Boc groups of **7** by HCl in MeOH and water afforded compound GdnDEG-SS in 76% yield. By reduction with dithiothreitol in water, GdnDEG-SH was successfully synthesized in 82% yield. Detailed synthetic procedures and characterization data of all the synthesized compounds by ^1^H and ^13^C nuclear magnetic resonance (NMR) and ESI-TOF or MALDI-TOF MS spectroscopic measurements are described in the Materials and Methods section.

### 2.2. Disulfide Bond Formation of RNase A

**Figure 3:**
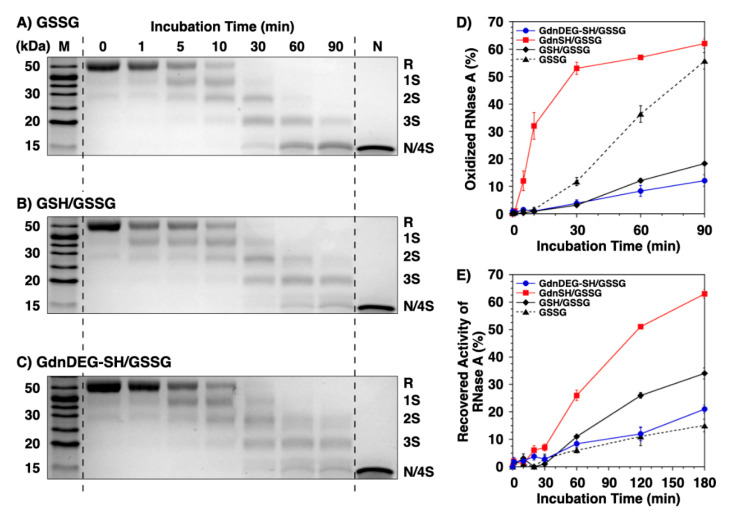
Time course analyses of RNase A oxidation by SDS-PAGE. SDS-PAGE gel images monitoring the oxidation of RNase A (8.0 μM) in the presence of (**A**) oxidized glutathione (GSSG), (**B**) GSH and GSSG, and (**C**) GdnDEG-SH and GSSG (thiol compounds: 1.0 mM; GSSG: 0.20 mM) in a buffer (50 mM Tris-HCl, 300 mM NaCl, pH 7.5). The oxidation reactions were quenched with maleimide PEG (Mw 2000) after 1, 5, 10, 30, 60, and 90 min incubation. The leftmost and rightmost lanes show the bands corresponding to the markers (M) and native RNase A (N), respectively. R and N/4S represent fully reduced and fully oxidized (a mixture of the native form and a folding intermediate with four disulfide bonds) RNase A, respectively. (**D**) Quantification of the relative band intensities of fully oxidized RNase A (8.0 μM) to the control (N) in the presence of mixtures of thiol (1.0 mM) and disulfide compounds (0.20 mM; blue filled circles: GdnDEG-SH/GSSG, red filled squares: GdnSH/GSSG [17], black filled diamonds: GSH/GSSG, and black filled triangles: GSSG only) quantified from SDS-PAGE analyses. (**E**) Recovered enzymatic activity of RNase A (8.0 μM) during incubation in the GdnDEG-SH/GSSG (blue filled circles), GdnSH/GSSG (black filled diamonds) [17], GSH/GSSG (black filled diamonds), and GSSG only (black filled triangles) systems (thiol compounds: 1.0 mM; disulfide compounds: 0.20 mM) in a buffer (50 mM Tris-HCl, 300 mM NaCl, pH 7.5). The activity was evaluated by spectroscopic monitoring of the hydrolysis of cytidine 2′:3′-cyclic monophosphate (cCMP) to 3′-CMP at 30 °C. Error bars indicate the means ± SEM of three independent experiments.

To study the effects of GdnDEG-SH on the progress of oxidative protein folding, we monitored the disulfide-bond formation of reduced and denatured ribonuclease (RNase) A. RNase A, that forms four disulfide bonds, i.e., C26–C84, C40–C95, C58–110, and C65–C72, is a representative protein for the oxidative folding studies [19]. During the folding process of RNase A, the reaction was quenched by addition of maleimide PEG (Mw 2000). Maleimide PEG couples with the thiol groups in RNase A to increase its mass, which results in decrease in the electrophoretic mobility in sodium dodecyl sulfate-polyacrylamide gel electrophoresis (SDS-PAGE) to separate bands of the fully reduced form (R), folding intermediates, and fully oxidized form (namely, a mixture of the native form and a nonnative form with four disulfide bonds, represented as N and 4S, respectively) of RNase A. In short, the SDS-PAGE assays monitor the progress of the disulfide bond formation as the mobility of bands. The disulfide bond formation of RNase A was triggered by addition of an oxidant such as oxidized glutathione (GSSG). In the SDS-PAGE assay of RNase A in the presence of GSSG, the band intensity of R decreased over the incubation time (Figure 3A). After 30 min incubation, the R band almost disappeared, while the N/4S band emerged. The intensity of the N/4S band increased over time, indicating progression of oxidation reaction. In the mixture of GSH and GSSG, the rate of the oxidation reaction decreased. Namely, the R band was visible even after 30 min incubation, and the color density of 3S band was higher than that of N/4S band after 90 min incubation (Figure 3B). A similar progression profile of the oxidation reaction was observed in the mixture of GdnDEG-SH and GSSG (Figure 3C). Quantitative analysis of N/4S formation of RNase A shows significant reduction in the oxidation rate in the GSH/GSSG and GdnDEG-SH/GSSG systems compared to that in the presence of GSSG only. As reported previously, GdnSH/GSSG system enhanced the oxidation reaction rate of reduced RNase A compared to GSH/GSSG system, indicating contrastive effects between GdnSH and GdnDEG-SH.

### 2.3. Enzymatic Activity Recovery of RNase A

To study the effect of GdnDEG-SH on the oxidative folding of RNase A to the native form, recovery of its enzymatic activity was monitored (Figure 3E). In the presence of GSSG as an oxidant, the enzymatic activity increased over time to reach 15% after 180 min incubation [18]. The recovered enzymatic activity of RNase A increased by addition of GSH and GdnSH in the presence of GSSG (34% and 63%, respectively) [18]. Here, addition of GdnDEG-SH did not prompt the folding reaction of RNase A to form the native form, and the profile of the enzymatic activity recovery in the GdnDEG-SH/GSSG system was analogous to that in the system of GSSG only.

### 2.4. Oxidative Folding of BPTI

**Figure 4:**
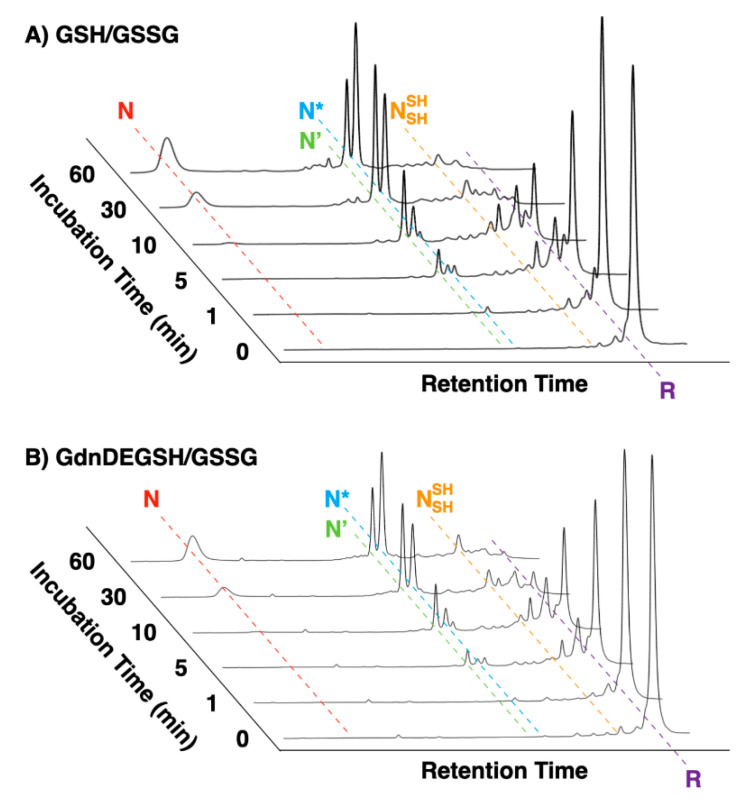
Time course reverse-phase HPLC analyses of oxidative folding of bovine pancreatic trypsin inhibitor (BPTI) (30 μM) in the presence of (**A**) GdnDEG-SH and GSSG and (**B**) GSH and GSSG (thiol compounds: 1.0 mM; GSSG: 0.20 mM). N and R represent native and reduced forms of BPTI, respectively. N’, N*, and $N_{\mathrm{SH}}^{\mathrm{SH}}$ represent folding intermediates of BPTI. Column: Tosoh Bioscience TSKgel Protein C4-300 (*φ*4.6 × 150 mm); eluent buffers: water (containing 0.05% trifluoroacetic acid (TFA)) and CH_3_CN (containing 0.05% TFA) with a linear gradient; flow rate: 1.0 mL min^−1^; detection wavelength: 229 nm; temperature: 25 °C.

Oxidative folding of bovine pancreatic trypsin inhibitor (BPTI) was investigated in the presence of the thiol compounds. BPTI contains three disulfide bonds, i.e., C5–C55, C14–C38, and C30–C51. In the folding pathway, it is known that BPTI forms some quasi-native intermediates such as N’, N*, and $N_{\mathrm{SH}}^{\mathrm{SH}}$ that can be separated by reverse-phase HPLC (RP-HPLC) for quantitative analysis [20]. These species act as a kinetical trap with native-like structures as evidenced by NMR [20]. Thus, oxidative folding assay by RP-HPLC provides structural insights into their folding intermediates. In a buffer containing Tris-HCl (50 mM, pH 7.5) and NaCl (300 mM), reduced and denatured BPTI (30 μM) was incubated in the presence of a thiol compound (1.0 mM) as a reductant and GSSG as an oxidant (0.20 mM) to allow for the folding to its native form. In the presence of GSH as the reductant, formation of the folding intermediates was observed within the initial 5 min incubation (Figure 4A). The fraction corresponding to the native form appeared after 10 min incubation and the yield of the native form was 24% after 60 min incubation. In the presence of GdnDEG-SH, an essentially similar change was observed, where the yield of the native form after 60 min was 19% along its on-path folding pathway. As we reported previously, the folding of BPTI was promoted in the presence of GdnSH to afford 51% yield of the native form after 60 min incubation [18]. Therefore, insertion of a diethylene glycol chain between the thiol and guanidyl groups significantly lowers the folding promotion function. RP-HPLC analyses also indicate different profiles of N’ and N* intermediates fractions in the presence of GdnDEG-SH and GdnSH, suggesting different influences of the reagents on the folding intermediates.

### 2.5. Characterization of GdnDEG-SH

By UV absorption spectroscopic measurements in buffers with variable pH values, p*K*_a_ of GdnDEG-SH was evaluated to be 9.28 ± 0.08. HPLC analyses monitoring the redox reactions with dithiothreitol indicated the redox potential *E*^0^′ of GdnDEG-SH to be −276 ± 0.4 mV. *E*^0^′ of GdnDEG-SH was lower than those of GdnSH (−237 ± 4 mV) [18] and GSH (−256 mV) [21], indicating that GdnDEG-SH is most reductive among these thiols. Meanwhile, p*K*_a_ of GdnDEG-SH was higher than that of GdnSH (8.86 ± 0.02) and GSH (9.15 ± 0.04). Therefore, the acidity of the thiol group of GdnDEG-SH is lower than GdnSH and GSH.

### 2.6. Aggregation Inhibition

**Figure 5:**
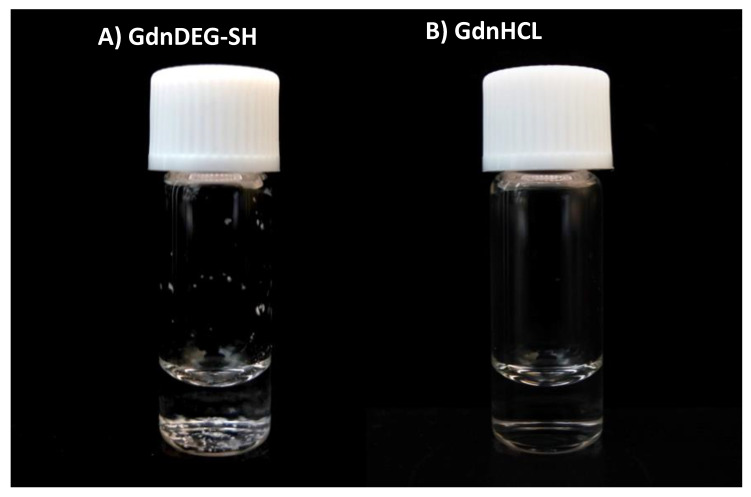
A photograph of a glass bottle containing lysozyme (20 μM) in the presence of (**A**) GdnDEG-SH (1.0 mM) and (**B**) guanidine hydrochloride (GdnHCl, 0.50 M) in a buffer (50 mM Tris-HCl, 300 mM NaCl, pH 7.5) after cooling from 96 to 25 °C. In (**A**), white precipitates are observed at the bottom of the glass bottle.

The aggregation inhibition effect of GdnDEG-SH was studied using lysozyme. Namely, a mixture of lysozyme (20 μM) and GdnDEG-SH (1.0 mM) in Tris-HCl buffer (50 mM, pH 7.5) containing NaCl (300 mM) was heated. After incubation at 96 °C for 20 min, white precipitates were observed (Figure 5A). In the presence of guanidine hydrochloride (GdnHCl, 0.50 M), lysozyme was solubilized even after the heat treatment (Figure 5B). Thus, GdnDEG-SH hardly showed aggregation suppression effect in the thermal denaturation condition of lysozyme.

## 3. Discussion

In an oxidative protein folding process, disulfide bond formation is one of the major factors to drive the reaction and control the pathway. Conversion of the disulfide bonds from nonnative to native pairs allows for enhancement of the yield of native form. Addition of thiol compounds with high nucleophilicity and oxidizability can facilitate shuffling of the disulfide bonds, which is effective for promotion of the oxidative protein folding [22,23].

In the folding processes of RNase A and BPTI, promotion effect of GdnDEG-SH was lower than that of GdnSH and rather similar to that of GSH. Coupling of a basic group such as guanidyl unit in the proximity of a thiol unit allows for increasing the acidity of the thiol, which is, therefore, advantageous to the folding promotion. In a previous study [24], it is reported that p*K*_a_ of the thiol group and solvent-accessible surface area of the positively charged amino moiety influence acceleration effects of thiol reagents possessing amino groups on disulfide-coupled protein folding. As demonstrated above, the acidity of GdnDEG-SH was lower than GdnSH and even than GSH. Thus, separation between the guanidyl and thiol units by insertion of a diethylene glycol chain significantly reduced the coupling effect to increase the thiol acidity likely due to extended distance, increased conformational flexibility, and/or altered polarity. Insertion of the diethylene glycol chain also lowered the redox potential of the thiol compound. Since GdnDEG-SH and GdnSH possess an identical basic unit (guanidyl unit), the reductive nature of GdnDEG-SH with lowered acidity likely resulted in the limited activity for the oxidative protein folding promotion.

PDI prevents proteins from aggregating and misfolding [25], indicating that an aggregation inhibition effect is an additional important property required for the folding promoting agents. Indeed, GdnSH and other thiol compounds with high folding promotion capabilities are functional to inhibit aggregation of denatured proteins [18,26,27,28]. In contrast, as shown in Figure 5A, GdnDEG-SH hardly showed aggregation suppression effect in the thermal denaturation condition of lysozyme, while GdnSH shows aggregation inhibition even at 1.0 mM [18]. This increased efficiency of the aggregation suppression is likely due to the high nucleophilicity of the thiol group in GdnSH, which allows for efficient localization of GdnSH onto the unfolded and folding-intermediate proteins to enhance the solubility. Thus, the lowered nucleophilicity of GdnDEG-SH likely resulted in reduced localization efficiency, which caused the insufficient aggregation inhibition property.

## 4. Materials and Methods

### 4.1. General

Nuclear magnetic resonance (NMR) spectra were recorded on a JNM-ECX 400 spectrometer (^1^H/400 MHz, ^13^C/100 MHz) of JEOL (Tokyo, Japan), where the chemical shifts were determined with respect to a ^1^H signal corresponding to the nondeuterated solvent and a ^13^C signal corresponding to the solvent as an internal standard (^1^H-NMR: 7.24 ppm for CDCl_3_, 4.67 ppm for D_2_O, 3.29 ppm for CD_3_OD; ^13^C-NMR: 77.16 ppm for CDCl_3_, 47.68 ppm for CD_3_OD). Matrix-assisted laser desorption/ionization time-of-flight (MALDI-TOF MS) was performed on autoflex speed spectrometer of Bruker (Bremen, Germany). Electrospray ionization (HR ESI) TOF MS spectra were recorded on micrOTOF-Q II-S1 of Bruker with MeOH as a solvent. Analytical thin layer chromatography (TLC) was performed on precoated, glass-backed silica gel Merck 60 F254. Visualization of the developed chromatogram was performed by UV absorbance, Hanessian’s stain or iodine. UV absorption spectra and temperature-dependent transmittance changes were recorded on V-650 UV–VIS spectrophotometer of JASCO (Tokyo, Japan) or U-3310 spectrophotometer of Hitachi High-Technologies (Tokyo, Japan). Reversed-phase high-performance liquid chromatography (RP-HPLC) for BPTI assay was conducted with GL7400 HPLC system of GL Sciences (Tokyo, Japan) using TSKgel Protein C4-300 column of Tosoh Bioscience (*φ*4.6 × 150 mm, Tokyo, Japan). RP-HPLC for redox potential measurement was conducted with HPLC system of JASCO (Tokyo, Japan) using YMC Triart C18 column (*φ*4.6 × 250 mm, Tokyo, Japan).

### 4.2. Materials

Deuterated solvents and di-*tert*-butyl decarbonate were purchased from Kanto Chemicals (Tokyo, Japan). maleimide-PEG (Mw 2000) was purchased from NOF America Corporation (White Plains, NY, USA). Acetonitrile, 35% hydrochloric acid, sodium hydroxide, tosyl chloride, sodium sulfate, potassium carbonate, ammonium chloride, sodium iodide, 30% hydrogen peroxide, sodium hydrogen carbonate, trifluoroacetic acid, and 2-propanol were purchased from Kishida Chemical (Tokyo, Japan). Coomassie brilliant blue, 5,5′-dithiobis(2-nitrobenzoic Acid) (DTNB), 1,4-dithiothreitol (DTT), l-glutathione oxidized (GSSG), l-glutathione reduced (GSH), and guanidine hydrochloride (GdnHCl) were purchased from Nacalai Tesque (Kyoto, Japan). α-Cyano-4-hydroxycinnamic acid, cytidine 2′:3′-cyclic monophosphate (cCMP) monosodium salt, triethylamine (Et_3_N), and ribonuclease A (RNase A) from bovine pancreas were purchased from Sigma-Aldrich (St. Louis, MO, USA). *N*,*N*′-Bis(*tert*-butoxycarbonyl)-1*H*-pyrazole-1-carboxamidine, 1,1′-carbonyldiimidazole, 2-(2-aminoethoxy)ethanol, potassium thioacetate, and gentisic acid were purchased from Tokyo Chemical Industry (Tokyo, Japan). Bovine pancreatic trypsin inhibitor (BPTI) was purchased from Takara Bio (Shiga, Japan). Hen egg white lysozyme was purchased from Fujifilm Wako Pure Chemical (Osaka, Japan). Dry *N*,*N*-dimethylformamide (DMF), dry CH_2_Cl_2_, and dry tetrahydrofuran (THF) were purchased from Kanto Chemicals. Column chromatography was carried out with Silica Gel 60 (spherical, neutral, particle size: 63–210 μm) purchased from Kanto Chemicals. Deionized water (filtered through a 0.22 μm membrane filter, >18.2 MΩ cm) was purified in Purelab DV35 of ELGA (Buckinghamshire, UK) and a Milli-Q system of Merck Millipore (Burlington, MA, USA).

### 4.3. Determination of Thiol Concentration for Oxidative Protein Folding Assay

To a solution of thiol (40 mM) in 10 mM HCl aqueous solution, Ellman’s reagent [5,5-dithio-bis-(2-nitrobenzoic acid), 0.50 mM] [29] was added. Concentration of the thiol group was determined by measuring the absorbance at 412 nm at 30 °C with U-3310 spectrophotometer prior to its use for the oxidative protein folding assay. Amounts of free thiol groups were determined by Ellman’s reagent. Each amount includes both the free thiol of 1.0 mM reducing agent (GdnDEG-SH) and 8.0 μM reduced substrate, RNase A.

### 4.4. Preparation of Reduced and Denatured RNase A

RNase A was dissolved in a buffer (200 mM Tris-HCl, pH 8.7) containing 6.0 M GdnHCl and 100 mM DTT and incubated for 2 h at 25 °C. The resulting sample was dialyzed by 10 mM HCl aq. to remove the denaturing and reducing reagents.

### 4.5. RNase A Refolding Assay

Fully reduced and denatured RNase A (8.0 μM) was incubated for 3 h at 30 °C in the presence of 200 μM GSSG and 1.0 mM reducing agent in a buffer (50 mM Tris-HCl, 300 mM NaCl, pH 7.5) [30]. At selected time points during this incubation, aliquots (50 μL each) were taken from the reaction solution, which were immediately added to a buffer (150 μL, 50 mM Tris-HCl, 300 mM NaCl, pH 7.5) containing cCMP (final concentration of cCMP = 0.60 mM), followed by the measurement of the linear increase in absorbance at 284 nm at 30 °C with U-3310 spectrophotometer [18,31]. Values represent means ± SEM from three independent experiments.

### 4.6. Gel Shift Assay of RNase A Disulfide Bond Formation

Oxidative folding of RNase A (8.0 μM) was carried out in a buffer (50 mM Tris-HCl, 300 mM NaCl, pH 7.5) containing 200 μM GSSG with/without 1.0 mM reducing agent. At selected time points, free thiols were blocked by the addition of Laemmli’s 4 × SDS-loading buffer [32] containing 10 mM maleimide-PEG (Mw 2000). Redox states of RNase A were separated by nonreducing 14% SDS-PAGE using WIDE RANGE gel (Nacalai Tesque). Proteins were detected by Coomassie brilliant blue G250 staining. The band intensities were analyzed by a ChemiDoc Touch imaging system and Image Lab (Bio-Rad).

### 4.7. BPTI Folding Assay

Reduction and denaturation of BPTI was carried out as described previously [25]. Fully reduced and denatured BPTI (30 μM) was incubated at 30 °C in the presence of 200 μM GSSG and 1.0 mM reducing agent in a buffer (50 mM Tris-HCl, 300 mM NaCl, pH 7.5), where the buffer was degassed by flushing N_2_ prior to use. At selected time points, the reaction was quenched by adding an equal volume of 1 M HCl aq., which was then analyzed by RP-HPLC at a flow rate of 1.0 mL min^−1^ monitoring at 229 nm with a linear gradient elution (solvent A: 0.05% trifluoroacetic acid in water and solvent B: 0.05% trifluoroacetic acid in acetonitrile; percentages of solvent A: 95% at 0 min, 80% at 15 min, and 30% at 115 min). The molecular mass values of the folding intermediates were determined by MALDI-TOF MS in a linear positive-ion mode using α-cyano-4-hydroxycinnamic acid (Sigma-Aldrich) as the matrix. The molecular mass was calculated using Protein-Prospector web server [33].

### 4.8. Determination of Thiol pK_a_ Values

Stock solutions of citric buffer (sodium citrate and HCl for pH 2.0–4.0), phosphate buffer (Na_2_HPO_4_ and KH_2_PO_4_ for pH 5.0–8.0), and borate buffers (Na_2_B_4_O_7_ and HCl for pH 8.5–9.0 and Na_2_B_4_O_7_ and NaOH for pH 10.0–12.0) were prepared, and these buffers were degassed with N_2_ for 1 h immediately prior to use. A stock solution of a thiol compound in degassed water (5.0 mM) were then prepared. Immediately after the aqueous solution of the thiol compound (20 μL) and a buffer (1.98 mL) were combined in a 1-cm thick quartz cuvette, the UV absorption spectrum of the sample was measured. The pH value of the sample was measured by a HORIBA pH meter (9618S-10D), which had been calibrated prior to use with pH 4.01, 6.86, and 9.18 standard solutions (HORIBA 101-S). Absorbance at 240 nm was plotted in the function of the pH values, and the p*K*_a_ value of the thiol compound was calculated with KaleidaGraph software (version 4.5.0) by a curve fitting analysis using the following equation:

y = a + (m1)/(m2 × 10^−x^ + 1); m1 = b; m2 = 1000, (1)

where a is the absorbance below the pH 3 and b is the difference of absorbances above the pH 11 and below the pH 3, and curve fitting calculation provides m2 = p*K*_a_. For all analyses, *r*^2^ values were higher than 0.99.

### 4.9. Redox Potential E^0^′ Measurements

*E*^0^′ value of a thiol compound was determined by following the protocol described in a previous paper [6]. Buffer (100 mM Tris-HCl, 1.0 mM EDTA, pH 7.0) was treated by bubbling high purity N_2_ for longer than 1 h prior to use. DTT^red^ (60 μM, 4.5 mL) in the buffer was added to a disulfide (GdnSS or UreaSS, 60 μM, 4.5 mL) in the buffer under N_2_, which was stirred at 25 ± 0.1 °C for 24 h. To quench the reaction, an aliquot of the reaction mixture (1 mL) was added to 1 M HCl aq. (200 μL), and the obtained sample solution was immediately analyzed by RP-HPLC (YMC Triart C18 column, *φ*4.6 × 250 mm). The column was equilibrated with water containing 0.1% TFA at a flow rate of 1.0 mL min^−1^. The RP-HPLC analysis was conducted with water containing 0.1% TFA (eluent A) and CH_3_CN containing 0.1% TFA (eluent B) with a linear gradient (percentage of eluent B: 0% in 0–8 min, 0–6% in 8–15 min, 6–10% in 15–30 min). The concentrations of the species at equilibrium were calculated from the observed peak areas and corresponding calibration curves.

The equilibrium constant *K*_eq_ for the reaction (Equation (2)), described as Equation (3), was determined by averaging three times of individual experiments following the above procedure.

Disulfide + DTT^red^ ⇄ 2Thiol + DTT^ox^ (2)

(3) $$K_{\mathrm{eq}}=\frac{{\left[\mathrm{Thiol}\right]}^{2}\left[{\mathrm{DTT}}^{\mathrm{ox}}\right]}{\left[\mathrm{Disulfide}\right]\left[{\mathrm{DTT}}^{\mathrm{red}}\right]}$$

The redox potential *E*^0^′ was calculated by the Nernst’s equation (Equation (4))

(4) $$E^{0\prime }={E^{0\prime }}_{\mathrm{DTT}}+\frac{RT}{nF}\;\ln\;K_{\mathrm{eq}},$$

where *n* is the number of transferred electrons (*n* = 2), *F* is Faraday’s constant (96,500 C mol^−1^), *R* is the universal gas constant (8.314 J K^−1^ mol^−1^), *T* is the temperature (298 K), and *E*^0^′_DTT_ is the redox potential of DTT (−327 mV).

### 4.10. Aggregation Inhibition Assay

Lysozyme (20 μM) was dissolved in Tris-HCl buffer (50 mM, pH 7.5) containing NaCl (300 mM) and an additive, and the mixture was incubated at 96 °C for 20 min followed by air cooling to room temperature.

### 4.11. Synthesis of ***1***

To a dry EtOH solution (31 mL) of 2-(2-aminoethoxy)ethanol (1.52 g, 14.5 mmol), di-*tert*-butyl decarbonate (3.28 g, 15.0 mmol) was added at 0 °C under N_2_. After being stirred for 4 h at 25 °C, CH_2_Cl_2_ (100 mL) was added to the reaction mixture, and the mixture was washed with water (100 mL, twice) and brine (50 mL, once). The organic extract was dried over anhydrous Na_2_SO_4_ and filtered off from insoluble substances. The filtrate was evaporated to dryness under reduced pressure at 30 °C, and the residue was chromatographed on silica gel (Silica Gel 60) with CH_2_Cl_2_/MeOH (100/0 to 90/10 *v*/*v*) to allow isolation of 1 (2.81 g, 13.7 mmol) in 94% yield.

TLC *R_f_* (Merck 60 F254, CH_2_Cl_2_/MeOH = 90/10 *v*/*v*): 0.56; ^1^H-NMR (400 MHz, CDCl_3_, 25 °C): *δ* = 5.18 (brs, 1H), 3.73 (brs, 2H), 3.59–3.54 (m, 4H), 3.33 (q, *J* = 5.0 Hz, 2H), 2.84 (brs, 1H), 1.45 (s, 9H) ppm; ^13^C-NMR (100 MHz, CDCl_3_, 25 °C): *δ* = 156.24, 79.41, 72.33, 70.37, 61.70, 40.44, 28.46 ppm; MALDI-TOF MS (gentisic acid, positive mode): *m*/*z* = 228.132 (calculated *m*/*z* on the basis of the monoisotopic mass of C_9_H_19_NNaO_4_ [M + Na]^+^ = 228.121).

### 4.12. Synthesis of ***2***

To a dry THF solution (31 mL) of **1** (2.68 g, 13.1 mmol), 15% NaOH aq. (10 mL) and a THF solution (10 mL) of TsCl (2.94 g, 15.4 mmol) were added dropwise at 0 °C under N_2_. After being stirred for 12 h at 25 °C, water (100 mL) was added to the reaction mixture. The resulting mixture was extracted with CH_2_Cl_2_ (50 mL, three times). The collected organic extract was washed with brine (50 mL, once), dried over anhydrous Na_2_SO_4_, and filtered off from insoluble substances. The filtrate was evaporated to dryness under reduced pressure at 30 °C to allow isolation of **2** (4.06 g, 11.3 mmol) in 86% yield.

TLC *R_f_* (Merck 60 F254, CH_2_Cl_2_): 0.25; ^1^H-NMR (400 MHz, CDCl_3_, 25 °C): *δ* = 7.80 (d, *J* = 8.7 Hz, 2H), 7.36 (d, *J* = 8.7 Hz, 2H), 4.86 (brs, 1H), 4.18–4.15 (m, 2H), 3.63 (t, *J* = 4.6 Hz, 2H), 3.45 (t, *J* = 5.0 Hz, 2H), 3.24 (q, *J* = 5.0 Hz, 2H), 2.45 (s, 3H), 1.45 (s, 9H) ppm; ^13^C-NMR (100 MHz, CDCl_3_, 25 °C): *δ* = 156.24, 144.98, 133.09, 129.91, 128.01, 79.36, 70.40, 69.18, 68.41, 40.29, 28.46, 21.68 ppm; MALDI-TOF MS (gentisic acid, positive mode): *m*/*z* = 382.114 (calculated *m*/*z* on the basis of the monoisotopic mass of C_16_H_25_NNaO_6_S [M + Na]^+^ = 382.130).

### 4.13. Synthesis of ***3***

To a dry DMF solution (35 mL) of **2** (3.98 g, 11.1 mmol), AcSK (4.03 g, 35.3 mmol) was added at 25 °C under N_2_. After being stirred for 13 h at 90 °C, the reaction mixture was cooled to 25 °C. To the resulting mixture, water (300 mL) was added, and the mixture was extracted with CH_2_Cl_2_ (100 mL, four times). The collected organic extract was washed with brine (100 mL, four times), dried over anhydrous Na_2_SO_4_, and filtered off from insoluble substances. The filtrate was evaporated to dryness under reduced pressure at 30 °C, and the residue was chromatographed on silica gel (Silica Gel 60) with AcOEt/hexane (20/80 *v*/*v*) to allow isolation of **3** (2.69 g, 10.2 mmol) in 92% yield.

TLC *R_f_* (Merck 60 F254, EtOAc/hexane = 50/50 *v*/*v*): 0.54; ^1^H-NMR (400 MHz, CDCl_3_, 25 °C): *δ* = 4.87 (brs, 1H), 3.57 (t, *J* = 6.4 Hz, 2H), 3.51 (t, *J* = 5.0 Hz, 2H), 3.30 (q, *J* = 5.0 Hz, 2H), 3.08 (t, *J* = 6.4 Hz, 2H), 2.35 (s, 3H), 1.45 (s, 9H) ppm; ^13^C-NMR (100 MHz, CDCl_3_, 25 °C): *δ* = 195.46, 155.99, 79.36, 69.99, 69.53, 40.42, 30.62, 28.95, 28.48 ppm; MALDI-TOF MS (gentisic acid, positive mode): *m*/*z* = 286.113 (calculated *m*/*z* on the basis of the monoisotopic mass of C_11_H_21_NNaO_4_S [M + Na]^+^ = 286.109).

### 4.14. Synthesis of ***4***

To a dry EtOH solution (19 mL) of **3** (2.69 g, 10.2 mmol), K_2_CO_3_ (3.54 g, 25.6 mmol) was added at 25 °C under N_2_. After being stirred for 22 h at 25 °C, the reaction mixture was neutralized with saturated NH_4_Cl aq. To the mixture, water (100 mL) was added, and the mixture was extracted with AcOEt (50 mL, three times). The collected organic extract was dried over anhydrous Na_2_SO_4_, and filtered off from insoluble substances. The filtrate was evaporated to dryness under reduced pressure at 30 °C, and the residue was chromatographed on silica gel (Silica Gel 60) with AcOEt/MeOH (20/80 *v*/*v*) to allow isolation of **4** (948 mg, 4.28 mmol) in 42% yield.

TLC *R_f_* (Merck 60 F254, EtOAc/hexane = 20/80 *v*/*v*): 0.28; ^1^H-NMR (400 MHz, CDCl_3_, 25 °C): *δ* = 4.90 (brs, 1H), 3.59 (t, *J* = 6.4 Hz, 2H), 3.52 (t, *J* = 5.0 Hz, 2H), 3.32 (q, *J* = 5.0 Hz, 2H), 2.69 (dt, *J* = 7.8, 8.2 Hz, 2H), 1.55 (t, *J* = 8.2 Hz, 2H), 1.45 (s, 9H) ppm; ^13^C-NMR (100 MHz, CDCl_3_, 25 °C): *δ* = 155.91, 78.98, 72.42, 69.73, 40.26, 28.34, 24.11 ppm; MALDI-TOF MS (gentisic acid, positive mode): *m*/*z* = 244.098 (calculated *m*/*z* on the basis of the monoisotopic mass of C_9_H_19_NNaO_3_S [M + Na]^+^ = 244.099).

### 4.15. Synthesis of ***5***

To an EtOAc solution (11 mL) of **4** (942 mg, 4.26 mmol), NaI (6.0 mg, 40 μmol) and 30% H_2_O_2_ aq. (480 μL) were added at 25 °C under air. After being stirred for 1 h at 25 °C, to the reaction mixture, saturated NaHCO_3_ aq. (100 mL) was added, and the mixture was extracted with EtOAc (50 mL, three times). The collected organic extract was washed with brine (50 mL, once), dried over anhydrous Na_2_SO_4_, and filtered off from insoluble substances. The filtrate was evaporated to dryness under reduced pressure at 25 °C to allow isolation of **5** (753 mg, 1.71 mmol) in 80% yield.

TLC *R_f_* (Merck 60 F254, EtOAc/hexane = 20/80 *v*/*v*): 0.32; ^1^H-NMR (400 MHz, CDCl_3_, 25 °C): *δ* = 5.13 (brs, 1H), 3.71 (t, *J* = 6.4 Hz, 2H), 3.53 (t, *J* = 5.0 Hz, 2H), 3.30 (q, *J* = 5.0 Hz, 2H), 2.88 (t, *J* = 6.4 Hz, 2H), 1.44 (s, 18H) ppm; ^13^C-NMR (100 MHz, CDCl_3_, 25 °C): *δ* = 155.94, 79.14, 69.98, 69.14, 40.36, 38.57, 28.42 ppm; MALDI-TOF MS (gentisic acid, positive mode): *m*/*z* = 463.192 (calculated *m*/*z* on the basis of the monoisotopic mass of C_18_H_36_N_2_NaO_2_S_2_ [M + Na]^+^ = 463.191).

### 4.16. Synthesis of ***6***

To a CH_2_Cl_2_ solution (11 mL) of **5** (752 mg, 1.71 mmol), TFA (2.5 mL) was added at 0 °C under N_2_. After being stirred for 2 h at 0 °C, the reaction mixture was evaporated to dryness under reduced pressure at 25 °C. To the residue, water (50 mL) was added, and the mixture was washed with CH_2_Cl_2_ (50 mL, three times). The aqueous extract was evaporated to dryness under reduced pressure to allow isolation of **6** (738 mg, 1.69 mmol) in 99% yield.

TLC *R_f_* (Merck 60 F254, EtOAc): 0.05; ^1^H-NMR (400 MHz, CD_3_OD, 25 °C): *δ* = 3.79 (t, *J* = 6.4 Hz, 4H), 3.71 (t, *J* = 5.0 Hz, 4H), 3.13 (t, *J* = 5.0 Hz, 4H), 2.94 (t, *J* = 6.4 Hz, 4H) ppm; ^13^C-NMR (100 MHz, CD_3_OD, 25 °C): *δ* = 69.10, 66.24, 39.25, 37.55 ppm; MALDI-TOF MS (gentisic acid, positive mode): *m*/*z* = 241.111 (calculated *m*/*z* on the basis of the monoisotopic mass of C_8_H_21_N_2_O_2_S_2_ [M + Na]^+^ = 241.104).

### 4.17. Synthesis of ***7***

To a dry DMF solution (15 mL) of **6** (770 mg, 1.77 mmol), *N*,*N*′-bis(*tert*-butoxycarbonyl)-1*H*-pyrazole-1-carboxamidine (1.209 g, 3.898 mmol) and triethylamine (990 μL, 7.09 mmol) were added at 25 °C under N_2_. After being stirred for 16 h at 25 °C, to the reaction mixture, CH_2_Cl_2_ (30 mL) was added, and the mixture was washed with water (100 mL, three times) and brine (50 mL, once). The organic extract was dried over anhydrous Na_2_SO_4_ and filtered off from insoluble substances. The filtrate was evaporated to dryness under reduced pressure at 30 °C, and the residue was chromatographed on silica gel (Silica Gel 60) with AcOEt/hexane (20/80 to 50/50 *v*/*v*) to allow isolation of **7** (846 mg, 1.16 mmol) in 66% yield.

TLC *R_f_* (Merck 60 F254, EtOAc/hexane = 20/80 *v*/*v*): 0.15; ^1^H-NMR (400 MHz, CDCl_3_, 25 °C): *δ* = 11.47 (brs, 2H), 8.62 (brs, 2H), 3.74 (t, *J* = 6.4 Hz, 4H), 3.61 (m, 8H), 2.90 (t, *J* = 6.4 Hz, 4H), 1.50 (s, 36H) ppm; ^13^C-NMR (100 MHz, CDCl_3_, 25 °C): *δ* = 163.61, 156.37, 153.08, 83.10, 79.35, 69.40, 69.16, 40.66, 38.45, 28.37, 28.16 ppm; ESI-TOF MS (MeOH, positive mode): *m*/*z* = 725.3609 (calculated *m*/*z* on the basis of the monoisotopic mass of C_30_H_57_N_6_O_10_S_2_ [M + Na]^+^ = 725.3578).

### 4.18. Synthesis of GdnDEGSS

To a MeOH solution (3 mL) of **7** (198 mg, 0.272 mmol), 1 M HCl aq. (10 mL) was added at 25 °C under air. After being stirred for 2 h at 25 °C, the reaction mixture was evaporated to dryness under reduced pressure at 25 °C. To the residue, water (10 mL) was added, and the mixture was washed with CH_2_Cl_2_ (30 mL, three times). The aqueous extract was evaporated to dryness under reduced pressure to allow isolation of GdnDEGSS (86 mg, 0.22 mmol) in 76% yield.

TLC *R_f_* (Merck 60 F254, MeOH): 0.01; ^1^H-NMR (400 MHz, D_2_O, 25 °C): *δ* = 3.73 (t, *J* = 6.0 Hz, 4H), 3.60 (t, *J* = 5.0 Hz, 4H), 3.31 (t, *J* = 5.0 Hz, 4H), 2.85 (t, *J* = 6.0 Hz, 4H) ppm; ^13^C-NMR (100 MHz, D_2_O, 25 °C): *δ* = 157.37, 68.77, 68.34, 41.30, 37.43 ppm; ESI-TOF MS (MeOH, positive mode): *m*/*z* = 325.1487 (calculated *m*/*z* on the basis of the monoisotopic mass of C_10_H_25_N_6_O_2_S_2_ [M + H]^+^ = 325.1480).

### 4.19. Synthesis of GdnDEG-SH

To a degassed aqueous solution (10 mL) of GdnDEGSS (86 mg, 0.22 mmol), dithiothreitol (249 mg, 1.61 mmol) was added at 25 °C under N_2_. After being stirred for 6 h at 25 °C, to the reaction mixture, water (10 mL) was added, and the mixture was washed with a mixture of 2-propanol and CHCl_3_ (10/50 *v*/*v*, 20 mL, four times). The aqueous extract was evaporated to dryness under reduced pressure to allow isolation of GdnDEGSH (71 mg, 0.35 mmol) in 82% yield.

^1^H-NMR (400 MHz, D_2_O, 25 °C): *δ* = 3.58–3.55 (m, 4H), 3.29 (t, *J* = 5.0 Hz, 2H), 2.61 (t, *J* = 6.0 Hz, 2H) ppm; ^13^C-NMR (100 MHz, D_2_O, 25 °C): *δ* = 72.22, 68.54, 68.34, 41.21, 23.24 ppm; ESI-TOF MS (MeOH, positive mode): *m*/*z* = 164.0859 (calculated *m*/*z* on the basis of the monoisotopic mass of C_5_H_14_N_3_OS [M + H]^+^ =164.0858.

## 5. Conclusions

In this study, we investigated the effects to insert a diethylene glycol linkage between thiol and guanidyl units on oxidative protein folding reactions. Compared to a conjugate between thiol and guanidyl units with close proximity, the separated conjugate by the linkage showed lower promotion effects on the oxidative folding reactions of RNase A and BPTI. A reductive nature with weaker acidity of the thiol group in the separated conjugate and its insufficient aggregation inhibition effect likely resulted in the limited promotion effects of the oxidative protein folding processes. Hence, the distance between the thiol and basic units such as guanidyl group is an important factor to develop functional oxidative protein folding promotors.

## Data Availability

The authors declare that the data that support the findings of this study are available within the paper. All other information is available from the corresponding authors upon reasonable request. Samples of the compounds are not available from the authors.
